# Abusive Supervision and Job Dissatisfaction: The Moderating Effects of Feedback Avoidance and Critical Thinking

**DOI:** 10.3389/fpsyg.2017.00496

**Published:** 2017-03-31

**Authors:** Jing Qian, Baihe Song, Bin Wang

**Affiliations:** Business School, Beijing Normal UniversityBeijing, China

**Keywords:** abusive supervision, job dissatisfaction, feedback avoidance, critical thinking, moderation

## Abstract

Although research on the antecedents of job dissatisfaction has been developed greatly, we know little about the role of abusive supervision in generating job dissatisfaction. The contingencies under which abusive supervision relates to employees’ job dissatisfaction are still unknown. The present study aimed to fill this research gap by empirically exploring the abusive supervision-job dissatisfaction relationship as well as examining the moderating roles of feedback avoidance and critical thinking on this relationship. We tested the hypotheses with data from a sample of 248 employees from a high-tech communications company in northern China and found that: (a) abusive supervision was positively related to job dissatisfaction; (b) the positive relationship was moderated by both employees’ feedback avoidance and critical thinking. We conclude by extracting the theoretical as well as practical contributions, along with a discussion of the promising directions for future research.

## Introduction

Job dissatisfaction is usually, but not necessarily an undesirable phenomenon for organizations (Zhou and George, 2001). Although researchers have linked job dissatisfaction to many negative outcomes in the workplace, such as employee turnover (e.g., Hom et al., 1992), it has been demonstrated that job dissatisfaction is related to some positive outcomes, such as employee creativity (e.g., Zhou and George, 2001). Recently, the economic crisis led to growing stress and severe mental health problems in the workplace, which would exacerbate employees’ job dissatisfaction (Mucci et al., 2016). Not surprisingly, given its importance and prevalence in organizations, identifying the antecedents of job dissatisfaction has attracted great interest from researchers (e.g., Amundsen and Martinsen, 2014; Arenas et al., 2015). Some scholars have focused their attention on the dynamic role of supervisors in decreasing employees’ job dissatisfaction. Previous studies focused mainly on the role of positive leadership (e.g., empowering leadership, Amundsen and Martinsen, 2014; transformational leadership, Top et al., 2015). However, the influence of “bad” leadership in generating job dissatisfaction is still a largely unknown area. To address this gap, our study aims to identify the role of the “dark” side of leaders in generating employees’ job dissatisfaction.

In the present study, we examine abusive supervision as a potential antecedent of job dissatisfaction. Abusive supervision refers to “subordinates’ perceptions of the extent to which their supervisors engage in a sustained display of hostile verbal and non-verbal behaviors, excluding physical contact” (Tepper, 2000, p. 178). Previous studies have stressed the notion that this definition characterizes abusive supervision as a subjective assessment (e.g., Tepper, 2000; Martinko et al., 2013). Because the definition focuses on perceptions rather than behaviors, an employee may consider a leader’s behavior as abusive in one context, yet consider the same behavior as non-abusive in another context (Tepper, 2000). In the context of job dissatisfaction, abusive supervision may have the potential to escalate into more dangerous, destructive leadership (Avey et al., 2015), which in turn may make job dissatisfaction more serious. Indeed, as a typical manifestation of destructive leadership at work, abusive supervision seems to have natural links with employees’ job dissatisfaction (e.g., Breaux et al., 2008; Hobman et al., 2009; Bowling and Michel, 2011; Haggard et al., 2011; Kernan et al., 2011; Lin et al., 2013; Martinko et al., 2013). Although abusive supervision has been conceptually linked to job dissatisfaction (Tepper, 2000; Tepper et al., 2004), the empirical evidence is rare. To fill this research gap, the present study empirically examines the relation between abusive supervision and employees’ job dissatisfaction.

Studies identifying moderators in the relationship between abusive supervision and work outcomes have grown steadily (e.g., Ouyang et al., 2015; Li et al., 2016). This line of research is important, because identifying moderators that are capable of alleviating the negative influences of abusive supervision may help victims to survive (e.g., Whitman et al., 2014; Frieder et al., 2015). Conditioned by individual differences, employees’ reaction to the negative influence of abusive supervision varies (Mackey et al., 2013; Frieder et al., 2015). In fact, prior research has shown that employees who possess better resource management abilities will experience less negative attitudes (i.e., dissatisfaction, emotional exhaustion, turnover intentions, and reductions in work effort) generated by abusive supervision than those who are incapable of managing their resources (Frieder et al., 2015). When faced with abusive supervision, employees who have better social adaptability skills will perceive less job tension, emotional exhaustion, and job dissatisfaction (Mackey et al., 2013). This contingency perspective on abusive supervision thus urges researchers to take into account the individual differences when examining the negative influence of abusive supervision. We focused on feedback avoidance and critical thinking as potential moderators, because both variables are considered as important avoidance coping skills in dealing with stressful situations at work (e.g., Nandkeolyar et al., 2014; Whitman et al., 2014), and abusive supervision is likely to produce such stressful work scenarios (Bowling and Michel, 2011; Lian et al., 2014a; Frieder et al., 2015).

### Abusive Supervision and Job Dissatisfaction

Job dissatisfaction is a common manifestation of employees’ attitudes in the workplace (Zhou and George, 2001). Previous research on salespeople found that the nature and quality of the interaction between supervisors and subordinates could influence their job dissatisfaction significantly (e.g., Churchill et al., 1976; Jaworski and Kohli, 1991; Brown and Peterson, 1993). For example, arbitrary punishing behavior from superiors has been positively related to job dissatisfaction (Kohli, 1985; Schul et al., 1990).

Abusive supervision has broader effects on indices of employees’ attitudes (Tepper, 2000). Previous studies have shown that abusive supervision is conceptually associated with job dissatisfaction in a wide range of samples (Tepper et al., 2004; Breaux et al., 2008; Hobman et al., 2009; Bowling and Michel, 2011; Haggard et al., 2011; Kernan et al., 2011; Lin et al., 2013; Martinko et al., 2013). For instance, Tepper (2000) argued that employees who reported abusive supervision were more likely to experience lower levels of job satisfaction. Tepper et al.’s (2004) research on co-workers’ organizational citizenship behaviors (OCBs) also suggested that abused subordinates could experience less satisfaction. Recently, an empirical study has found that workplace bullying negatively relates to employees’ job satisfaction (Arenas et al., 2015). In the present study, we argue that abusive supervision as a common form of workplace bullying (Ouyang et al., 2015) could generate employees’ job dissatisfaction. This is because abusive supervision combines a variety of hostile actions, including emotional outbursts (Ashforth, 1994; Bies and Tripp, 1998), destructive and public criticism (Baron, 1993; Bies, 2000), and undermining social behaviors (Hoel et al., 1999; Duffy et al., 2002). According to social identity theory, when leaders treat their employees in a hostile way, employees’ sense of belonging to the organization will be reduced and employees are likely to feel depressed (e.g., Tajfel and Turner, 1985; Ouyang et al., 2015). Indeed, previous research has suggested that, compared with non-abused employees, employees under abusive supervision experience less favorable attitudes (Keashly et al., 1994; Ashforth, 1997; Keashly and Jagatic, 2000). Abusive supervision also constitutes a source of stress that is damaging to employees’ affective liking for their jobs.

Hypothesis 1: Abusive supervision is positively related to job dissatisfaction.

### The Moderating Role of Feedback Avoidance

Feedback avoidance refers to the intentional, proactive, and purposeful feedback management tactic involving “active behaviors directed at evading feedback” (Moss et al., 2009, p. 647). Recent research suggests that subordinates may engage in avoidant coping behaviors to deal with the stress caused by abusive leaders (Tepper et al., 2007; Whitman et al., 2014). According to the transactional theory of stress and coping strategies (Lazarus and Folkman, 1987), the nature of the stress-work outcomes relation depends on the coping strategies that subordinates implement (Nandkeolyar et al., 2014). In other words, the extent to which abusive leader as a workplace stressor can be detrimental to work outcomes depends on the coping strategies used by subordinates (Dijkstra et al., 2009; Nandkeolyar et al., 2014). Specifically, feedback avoidance may serve as a coping strategy to alleviate the stress or to protect an individual’s already limited resources (e.g., promotion and continued employment) by avoiding further abuse (Whitman et al., 2014). Indeed, previous studies have suggested that feedback avoidance can serve as a passive coping mechanism for abused subordinates to manage supervisory abuse and to avoid disciplinary reactions from their supervisors (Moss et al., 2003, 2009; Whitman et al., 2014). In the present study, we examine the moderating effect of feedback avoidance on the relationship between abusive supervision and employees’ job dissatisfaction.

Feedback avoidance represents a purposeful feedback management tactic (Moss et al., 2009). When dealing with abusive supervision, employees with high feedback avoidance may develop a safe distance, both physically and psychologically, by applying avoidance behaviors (Hess, 2000; Whitman et al., 2014). As a result, they could sense less discomfort related to abusive supervision. Indeed, research has suggested that employees who engage in more avoidance behaviors can alleviate the discomfort associated with threatening people and situations (Tepper et al., 2007). Thus, the effectiveness of abusive supervision in generating a job dissatisfaction level for employees with high feedback avoidance will be weaker.

In contrast, employees who engage in less avoidance behaviors fail to create a physical or psychological distance, meaning that they feel powerless in terms of coping with the undesirable relationships and negative consequences generated by their abusive supervisors. They are likely to perceive more discomfort which could in turn generate more job dissatisfaction. As such, we expect high feedback avoidance employees experience less job dissatisfaction from abusive supervision than low feedback avoidance employees do, thus the impact of abusive supervision on job dissatisfaction for high feedback avoidance employees is less effective.

Hypothesis 2: Feedback avoidance moderates the positive relationship between abusive supervision and job dissatisfaction in such a way that the relationship will be weaker when feedback avoidance is higher rather than lower.

### The Moderating Role of Critical Thinking

Critical thinking is a “purposeful, self-regulatory judgment which results in interpretation, analysis, evaluation, and inference, as well as explanation of the evidential, conceptual, methodological, criteriological, or contextual considerations upon which that judgment is based” (Facione, 1990, p. 2). It is a pervasive and self-rectifying personal phenomenon (Facione, 1990). With the emergence of knowledge workers, critical thinking as an important individual skill has been receiving great attention (e.g., Facione, 1990; Jiang and Yang, 2014; Samson, 2016). Previous studies have proposed that an ideal critical thinker is habitually inquisitive, well-informed, open-minded, flexible, fair-minded in evaluation, honest in facing personal biases, prudent in making judgments, willing to reconsider, orderly in complex matters, and diligent in seeking relevant information (Facione, 1990). Recent studies go further and suggest that critical thinking is related to employees’ creativity (Jiang and Yang, 2014) and leaders’ transformational behaviors (Godzyk, 2008). In light of the findings of previous studies, we extended this line of research by exploring critical thinking as a moderator of the relationship between abusive supervision and job dissatisfaction.

Critical thinking is a high-level thinking ability or thinking mode in terms of coping with problems, reasoning and finding solutions (Ruminski and Hanks, 1995). According to Lim (2011), the main components of critical thinking involve analysis, evaluation, and the construction of an argument. When dealing with abusive supervision, employees who are equipped with high levels with regard to critical thinking are more likely to have the capacity to understand, analyze, and resolve the problems generated by their supervisors’ abuse. They are well-informed, open-minded, flexible and are capable of formulating plausible hypotheses and drawing conclusions (Facione, 1990; Buraphadeja and Dawson, 2008). When experiencing supervisors’ abuse, they do not bear it in vain, but instead, they are able to identify and challenge the taken-for-granted assumptions and are willing to explore and practice alternative solutions (Vandsburger et al., 2010). As a result, the effectiveness of abusive supervision in generating job dissatisfaction levels for high critical-thinking employees will be weaker.

In contrast, employees who have a lower level of critical thinking see issues from an individual viewpoint. It is not easy for them to deeply analyze complex information, issues and problems (Celuch et al., 2009). When they experience their supervisors’ abuse, they are not flexible and are limited in terms of seeking relevant information and alternative solutions; they may instead accept this suffering in silence. Failing to apply proper cognitive strategies to cope with abusive supervision might result in a higher level of job dissatisfaction. The effectiveness of abusive supervision on enhancing a job dissatisfaction level thus will be higher when employees’ critical thinking is lower.

Hypothesis 3: Critical thinking moderates the positive relationship between abusive supervision and job dissatisfaction in such a way that the relationship will be weaker when employees’ critical thinking is higher rather than lower.

## Materials and Methods

### Sample and Procedure

Participants in our study were 320 full-time employees who came from a high-tech communications company in northern China. All participants took part in this survey voluntarily. The survey packets, including consent forms and survey questionnaires, were delivered to participants by one of the authors at a company-wide meeting. To ensure the participants’ confidentiality, we provided a return envelope with seal tape for participants to seal the finished questionnaire. Participants were asked to complete the survey questionnaires and to return them via the research box at the upcoming meeting 2 weeks later. During this period, we sent text messages on two occasions to the participants—3 days after the questionnaire was distributed and 1 day before the deadline. One was to encourage their involvement and the other was to remind them to return the finished questionnaire. This study was carried out in accordance with the Declaration of Helsinki and ethical guidelines and approved by the Human Research Ethics Committee (HREC) at the Australian National University. Written informed consent was obtained from all participants.

Of the 320 questionnaires distributed, a total of 248 surveys were returned in this study, for a response rate of 77.5%. The demographic data was as follows: the average age of the participants was 32.58 years old (*SD* = 8.28); 62.1% participants were men (*M* = 1.37; *SD* = 0.48); participants’ average organizational tenure was 6.31 years (*SD* = 3.99).

### Measures

#### Abusive Supervision

We measured abusive supervision using Tepper’s (2000) 15-items abusive supervision questionnaire. A sample item is, “My boss invades my privacy.” Respondents used a five-point response scale, where 1 was “I cannot remember him/her ever using this behavior with me,” 2 was “He/she very seldom uses this behavior with me,” 3 was “He/she occasionally uses this behavior with me,” 4 was “He/she uses this behavior moderately often with me,” and 5 was “He/she uses this behavior very often with me” (α = 0.73).

#### Job Dissatisfaction

We measured employees’ job dissatisfaction using Zhou and George’s (2001) three-items scale. A sample item is, “In general, I don’t like my job.” Response options ranged from 1, “strongly disagree” to 7, “strongly agree” (α = 0.91).

#### Feedback Avoidance

We measured feedback avoidance using Moss et al.’s (2003) six-items feedback avoiding behavior scale. A sample item is, “After performing poorly, I would try to avoid eye contact with my supervisor so that he/she didn’t start a conversation with me about my performance.” Response options ranged from 1, “extremely unlikely” to 7, “extremely likely” (α = 0.92).

#### Critical Thinking

We measured critical thinking using Jiang and Yang’s (2014) five-items critical thinking scale. A sample item is, “I try to find alternative solutions to the problem.” Response options ranged from 1, “strongly disagree” to 5, “strongly agree” (α = 0.70).

#### Control Variables

Previous studies have suggested that age, gender, and company tenure could affect responses to abusive supervision (e.g., Haggard et al., 2011; Martinko et al., 2013). For this reason, while respecting previous job dissatisfaction research (e.g., Zhou and George, 2001), we included three control variables (i.e., participants’ age, gender, and company tenure) for testing the hypotheses. Age and company tenure were measured by number of years. Gender was coded 0 for “female” and 1 for “male.”

## Results

### Confirmatory Factor Analyses

**Table 1** presents the CFA results. As shown, the baseline four-factor model fit the data well (χ^2^ = 568.4; df = 333; RMSEA = 0.05; CFI = 0.94; TLI = 0.95). Against this baseline four-factor model, we tested a null model, three three-factor models, and a two-factor model. As shown in **Table 1**, the baseline model (four factors) fits better than the null model as well as Model 1, Model 2, Model 3, and Model 4, providing evidence of the construct distinctiveness of abusive supervision, job dissatisfaction, feedback avoidance, and critical thinking.

**Table 1:** Comparison of measurement model.

Model	Factors	χ^2^	df	TLI	CFI	RMSEA
Null model		2125.9	340	0.55	0.60	0.15
Baseline model	Four factors	568.4	333	0.94	0.95	0.05
Model 1	Three factors: job dissatisfaction and feedback avoidance were combined into one factor	1360.5	337	0.74	0.77	0.11
Model 2	Three factors: abusive supervision and feedback avoidance were combined into one factor	1328.7	337	0.75	0.77	0.11
Model 3	Three factors: abusive supervision and job dissatisfaction were combined into one factor	1280	337	0.76	0.79	0.10
Model 4	Two factors: abusive supervision, job dissatisfaction and feedback avoidance were combined into one factor	1797.6	339	0.64	0.67	0.13

*CFI, comparative fit index; RMSEA, root-mean-square error of approximation; TLI, Tucker-Lewis index.*

### Descriptive Analyses

**Table 2** presents descriptive statistics for the correlations among the studied variables. As anticipated, abusive supervision was correlated positively with job dissatisfaction (*r* = 0.21, *p* < 0.01), thus providing preliminary support for Hypothesis 1.

**Table 2:** Means, standard deviations, reliabilities, and correlations among study variables.

	*M*	*SD*	1	2	3	4	5	6	7
(1) Age	32.58	8.28							
(2) Gender	1.37	0.48	0.00						
(3) Company tenure	6.30	3.98	0.48^∗∗^	–0.01	–				
(4) Abusive supervision	2.87	0.34	–0.13^∗^	0.01	–0.09	(0.73)			
(5) Job dissatisfaction	4.39	0.79	0.03	0.06	–0.03	0.21^∗∗^	(0.91)		
(6) Critical thinking	3.31	0.50	–0.02	–0.02	–0.01	0.05	0.11	(0.70)	
(7) Feedback avoidance	3.00	0.75	–0.09	0.14	–0.17	0.04	0.26^∗∗^	0.10	(0.92)

*N = 248. Cronbach’s alpha reliabilities are in parentheses on the diagonal. *^∗^p* < 0.05; ^∗∗^*p* < 0.01.*

### Hypothesis Testing

As shown in **Table 3**, abusive supervision was significantly and positively correlated with job dissatisfaction (β = 0.21, *p* < 0.01) after controlling for age, gender, and company tenure. Our first hypothesis was, therefore, fully supported.

**Table 3:** Results of regression analysis for moderation.

Variables	Step 1 β	Step 2 β	Step 3 β
Age	0.05	0.05	0.05
Gender	0.06	0.06	0.06
Company tenure	–0.06	–0.06	–0.06
Abusive supervision		0.21^∗∗^	0.21^∗∗^
Critical thinking		0.07	0.07
Feedback avoidance		0.25^∗∗^	0.25^∗∗^
Abusive supervision × Critical thinking			–0.28^∗^
Abusive supervision × Feedback avoidance			0.19^∗∗^
*R^2^*	0.01	0.12	0.23
*ΔR^2^*		0.11^∗∗^	0.11^∗∗^

*N = 248. Job dissatisfaction was the dependent variable. *^∗^p* < 0.05; ^∗^*^∗^p* < 0.01.*

**FIGURE 1:**
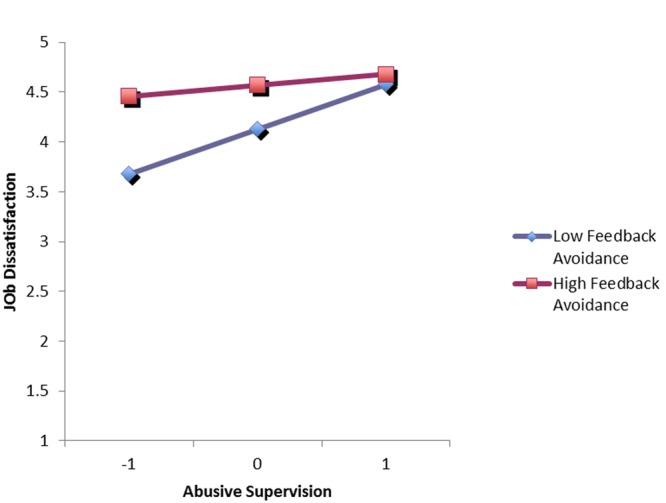
**Abusive supervision and job dissatisfaction by feedback avoidance**.

**FIGURE 2:**
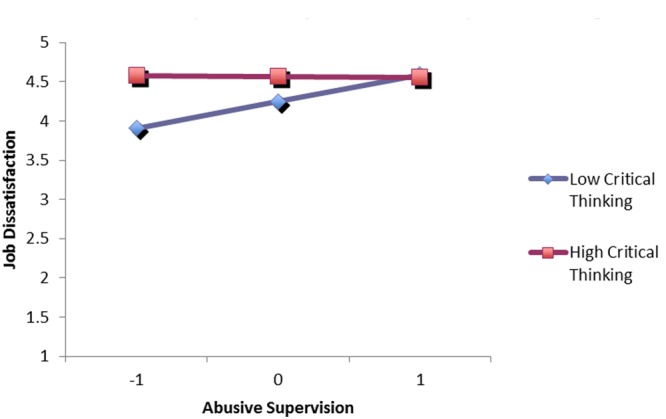
**Abusive supervision and job dissatisfaction by critical thinking**.

We tested our second and third hypotheses by entering the variables into the regression analysis at three hierarchical steps: (1) the control variables (i.e., participants’ age, gender, and company tenure); (2) abusive supervision, feedback avoidance, and critical thinking; (3) the two-way interaction terms of abusive supervision × feedback avoidance and abusive supervision × critical thinking. As shown in **Table 3**: (a) feedback avoidance significantly moderated the influence of abusive supervision on job dissatisfaction (β = -0.19, *p* < 0.01); (b) critical thinking significantly moderated the influence of abusive supervision on job dissatisfaction (β = -0.28, *p* < 0.01). To interpret the specific moderating effects in Hypotheses 2 and 3, we calculated regression equations for the relationship between abusive supervision and job dissatisfaction at high and low levels of feedback avoidance as well as at high and low levels of critical thinking. Following Cohen and Cohen (1983), we define high feedback avoidance and critical thinking as plus one standard deviation from the mean and define low feedback avoidance and critical thinking as minus one standard deviation from the mean. The results were reported in **Figures 1, 2**. As predicted: (a) the linear relationship between abusive supervision and job dissatisfaction was weaker for the high feedback avoidance employees and stronger for the low feedback avoidance employees; (b) the linear relationship between abusive supervision and job dissatisfaction was weaker for the high critical-thinking employees and stronger for the low critical-thinking employees. Thus, Hypotheses 2 and 3 were fully supported.

## Discussion

Although there has been a substantial amount of research on the antecedents of job dissatisfaction (e.g., Amundsen and Martinsen, 2014; Arenas et al., 2015), we know little about the relationship between abusive supervision and job dissatisfaction. Additionally, the contingencies under which abusive supervision relates to employees’ job dissatisfaction are still unknown. To this end, we proposed a model and found that: (1) abusive supervision is positively related to job dissatisfaction; (2) this positive relationship was moderated by employees’ feedback avoidance in such a way that the relationship will be weaker when feedback avoidance is higher rather than lower; (3) this positive relationship was moderated by employees’ critical thinking in such a way that the relationship will be weaker when employees’ critical thinking is higher rather than lower.

### Research Implications

The present research has a number of research implications regarding abusive supervision and job dissatisfaction. First, by exploring abusive supervision as an antecedent of employees’ job dissatisfaction, our findings encompassed a number of theoretical contributions to the growing research in identifying a leader’s role in generating employees’ levels of job dissatisfaction. By doing so, our study provided empirical support for previous studies that conceptually linked abusive supervision to job dissatisfaction (e.g., Tepper, 2000; Tepper et al., 2004; Breaux et al., 2008; Hobman et al., 2009; Bowling and Michel, 2011; Haggard et al., 2011; Kernan et al., 2011; Lin et al., 2013; Martinko et al., 2013).

Second, the present study hypothesized and found that individual difference variables of feedback avoidance and critical thinking could moderate the abusive supervision-job dissatisfaction relationship. Our findings echoed the call to advance the understanding of the contingency side of abusive supervision (Whitman et al., 2014). Previous studies have examined various boundary conditions of the influence of abusive supervision on employees, such as the moderating effect of political skill on the abusive supervision-employee burnout relationship (Li et al., 2016), and the moderating effect of subordinates’ gender on the abusive supervision-perceived insider status relationship (Ouyang et al., 2015). To the best of our knowledge, we made the first attempt to identify potential moderators on the abusive supervision-job dissatisfaction relationship.

Third, by identifying the moderating roles of feedback avoidance and critical thinking, our findings also contributed to the feedback avoidance and critical thinking literature. Previous studies have suggested avoidance as an ineffective coping strategy that may worsen the negative effects of stress (e.g., Carver and Connor-Smith, 2010; Nandkeolyar et al., 2014). For example, Nandkeolyar et al. (2014) suggested that the use of avoidance as a coping strategy might facilitate the negative relationship between abusive supervision and job performance. In the present study, we identified the positive moderating effects of feedback avoidance on alleviating the negative influences of abusive supervision. This finding is an important addition to the coping strategies literature, which could pay attention to the positive side of avoidance coping strategies (Skinner et al., 2003; Nandkeolyar et al., 2014).

Finally, critical thinking as an important individual skill in the workplace has received great attention in recent years (e.g., Facione, 1990; Jiang and Yang, 2014; Samson, 2016). However, the unique moderating effects that critical thinking may have on alleviating those “bad” leaders’ negative influences has not been theorized or empirically examined. Our findings addressed this research gap by examining critical thinking as a moderator on the relationship between abusive supervision and employees’ job dissatisfaction. By doing so, the present findings provided us with valuable insights into how to alleviate the negative influences of abusive supervision on employees’ job dissatisfaction.

### Practical Contribution

The present research provides some interesting implications for managerial practices. To begin with, our findings suggest that feedback avoidance is one of strategies to reduce abusive supervision’s impact on job dissatisfaction. Recently, scholars and practitioners alike have called for the promotion of seeking feedback at work in order to enhance performance (see the reviews by Ashforth and Anand, 2003; Anseel et al., 2015). However, employees’ attitudes and behaviors toward feedback involve more than one party. Given our findings that employees’ active behaviors directed at evading feedback serve as a coping strategy that abused subordinates rely on in order to deal with abusive supervision, organizations should first pay attention to avoiding or reducing managers’ abusive supervision through the recommended strategies, such as avoiding hiring individuals for managerial positions who are dispositionally inclined to have a narrow scope of justice or to execute hostile acts (Tepper et al., 2011). Organizations could also use justice training to help managers interact constructively with their subordinates (Tepper et al., 2011), mediate supervisor conflicts and promote strong leader-member relationships (Harris et al., 2011).

Additionally, after finding the neutralizing effects of critical thinking, organizations could pay extra attention to individual differences when selecting, recruiting and training employees. Specifically, organizations can select and recruit employees by testing their abilities related to critical thinking, such as evaluating their cognitive skills in interpretation, analysis, evaluation, inference, explanation and self-regulation, all of which are regarded as the core components of critical thinking (Facione, 1990). Previous studies also suggest that employees’ critical thinking can be developed (e.g., Abrami et al., 2008; Schneller and Brocato, 2011). For example, organizations can train their employees’ critical thinking by increasing their open-mindedness and ability to imagine alternative ways to assess and solve problems (e.g., Deal and Pittman, 2009).

### Limitations and Suggestions for Future Research

**FIGURE 3:**
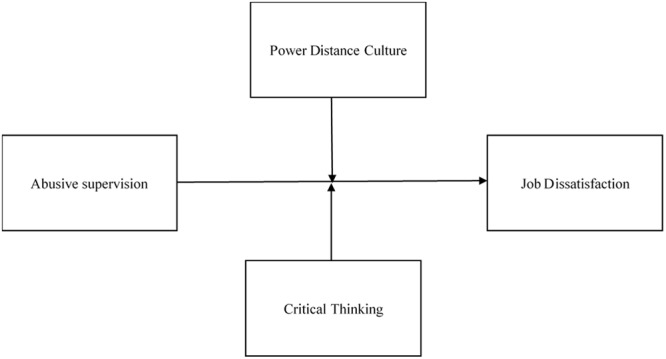
**A proposed model with power distance and critical thinking**.

In addition to the aforementioned contributions, the present study has some limitations that must be addressed. First, the data used in this research were only collected from one organization in China. Therefore, the extent to which the present findings are applicable to other types of organizations or cultures can only be speculated. Future researchers could investigate the generalisability of the present findings in different organizational settings and cultures. Second, the measures of abusive supervision and job dissatisfaction were derived from self-reports of respondents. Thus, the findings could be influenced by common method variance, response consistency effects, or other methodological issues common to self-report methods. This limitation is not uncommon in research on abusive supervision and job dissatisfaction (e.g., Zhou and George, 2001; Tepper, 2007; Martinko et al., 2013; Vogel et al., 2015; Li et al., 2016). A meaningful extension of this research is to include supervisors’ perceptions of abusive supervision and job dissatisfaction in an effort to reduce common method variance. Third, due to the nature of the cross-sectional design, the direction of causality cannot be ascertained from the present study. It is possible that abusive supervision is a reaction to employees’ job dissatisfaction (Liang et al., 2016). We suggest applying longitudinal or experimental designs to future studies. For instance, future researchers are encouraged to use a longitudinal cross-lagged panel design that allows for the simultaneous testing of bidirectional effects (Lian et al., 2014a,b). Using a longitudinal cross-lagged panel design, the same participants are asked to complete the same measures (i.e., abusive supervision and job dissatisfaction) at two separate points in time (separated by approximately 6 months). Temporal change is included precisely in the panel data, providing the foundation for assessing causation (Finkel, 1995). This allows researchers to test each variable’s predictive effect on the other while controlling for either variable’s existing levels (Lian et al., 2014b). In addition, measuring variables using videos of supervisors’ behaviors in experimental studies would be helpful for strengthening the conclusions of the present study (Martinko et al., 2013; Liang et al., 2016). According to Liang et al. (2016) experimental study, future researchers may instruct subordinates to complete a task visualizing their immediate supervisor. The participants are randomly assigned to one of two conditions: supervisor with abusive supervision or supervisor without abusive supervision. The subordinates then recall an interaction with the visualized supervisor. When the subordinates complete the recall task, they are asked to complete the survey questionnaires to rate their job dissatisfaction and the abusive supervision shown by the visualized supervisor. Fourth, in the present study, we only included participants’ age, gender, and company tenure as control variables for testing the hypotheses. However, previous studies have suggested that job dissatisfaction is strongly affected by personality traits and negative affectivity (Chen and Spector, 1991; Spector and O’Connell, 1994). We encourage future researchers to test the hypotheses by including more control variables (e.g., personality traits and negative affectivity). Finally, in the present study, we only focused on individual differences variables as moderators. Future research could investigate, for example, how situational variables moderate the relationship between abusive supervision and job dissatisfaction, or how individual variables interact with situational variables to exert joint moderating effects on this relationship. For instance, a multi-level model was proposed for future scholars to investigate the joint moderating effect of group power distance climate and the individual difference variable of critical thinking on the abusive supervision-job dissatisfaction relationship (see **Figure 3**). In this model, we suggested that the positive relationship between abusive supervision and job dissatisfaction will be stronger when the group has higher power distance climate; and this relationship will be the strongest when group power distance climate is higher while employees’ critical thinking is lower.

## Conclusion

Given its importance and prevalence in organizations, identifying the antecedents of job dissatisfaction has attracted great interest from researchers (e.g., Amundsen and Martinsen, 2014; Arenas et al., 2015). The present study examined abusive supervision as an antecedent of job dissatisfaction and it investigated feedback avoidance and critical thinking as moderators on the abusive supervision-job dissatisfaction relationship.

## Ethics Statement

All procedures performed in studies involving human participants were in accordance with the ethical standards of the institutional and/or national research committee and with the 1964 Helsinki declaration and its later amendments or comparable ethical standards with written informed consent from all subjects.

## Author Contributions

JQ, BS, and BW designed, drafted this study and collected the data. BS and BW contributed to the interpretation of the findings. All authors critically reviewed and approved the final version of this manuscript.

## Conflict of Interest Statement

The authors declare that the research was conducted in the absence of any commercial or financial relationships that could be construed as a potential conflict of interest.
